# Improving the External Quantum Efficiency of High-Power GaN-Based Flip-Chip LEDs by Using Sidewall Composite Reflective Micro Structure

**DOI:** 10.3390/mi12091073

**Published:** 2021-09-04

**Authors:** Liang Xu, Kaiping Fan, Huiqing Sun, Zhiyou Guo

**Affiliations:** 1 Institute of Semiconductor Science and Technology, South China Normal University, 55 Zhongshan Avenue, Tianhe District, Guangzhou 510631, China; xuliang11288@163.com (L.X.); sunhq@scnu.edu.cn (H.S.); 2 Foshan NationStar Semiconductor Co., Ltd., Foshan 528226, China; fankaiping@nationstar.com

**Keywords:** CRS-FCLEDs, light extraction efficiency, composite reflection microstructure

## Abstract

For high-power applications, it is important to improve the light extraction efficiency and light output of the vertical direction of LEDs. Flip-chip LEDs (FCLEDs) with an Ag/SiO_2_/distributed Bragg reflector/SiO_2_ composite reflection micro structure (CRS) were fabricated. Compared with the normal Ag-based FCLEDs, the light output power of the CRS-FCLEDs was increased by 6.3% at an operational current of 1500 mA, with the corresponding external quantum efficiency improved by 6.0%. Further investigation proved that the CRS structure exhibited higher reflectance compared with the commonly used Ag-mirror reflective structure, which originates from the increased reflective area in the sidewall and partial area of the n-GaN contact orifices. It exhibited markedly smaller optical degradation and thus higher device reliability as compared to normal Ag-based FCLED. Moreover, the light emission intensity distributions and far-field angular light emission measurements show that the CRS-FCLED has a strengthened light output in the vertical direction, which shows great potential for applications in high-power fields, such as headlamps for automobiles.

## 1. Introduction

With the development of semiconductor materials and packaging technology, the luminous flux of high-power white LEDs has rapidly improved. The application of LEDs in the automotive lighting field has gradually expanded, from signal indicators to current automotive headlights, reflecting the development trend that LEDs will become the mainstream light source in this field [1,2,3,4]. Flip-chip LEDs (FCLEDs) exhibit excellent heat dissipation performance and high light efficiency, and have been attracting great attention in the field of high-power LEDs. Forming a p-type ohmic contact electrode with high reflectance and low ohmic contact resistance is the key to realize high-efficiency FCLEDs [5,6,7]. However, high power LED lighting generally requires high current density and vertical light extraction efficiency owing to the dense packaging [8,9,10]. External quantum efficiency (EQE) is the ratio between the number of electrically injected carriers and externally observed photons. EQE is usually related to the reflectivity or reflective area of the mirror inside the chip and the surface light extraction structure such as the surface roughness and photonic crystals [11,12]. Internal Quantum Efficiency (IQE) is the ratio between the electrically injected carriers and the internally emitted photons. It mainly depends on the carrier injection efficiency and the compound efficiency of quantum well [13].

In order to improve the EQE, Ag-based reflective p-electrodes are widely used in FCLEDs owing to their superior electrical properties and high reflectivity in the visible wavelength range [14,15]. However, the Ag contact suffers from poor adhesion, inferior ohmic behavior, and thermal instability such as migration. Therefore, it is necessary to maintain a sufficient distance between the Ag mirror and a PN junction in the LED chip design, and cover it with a TiW barrier diffusion layer [16,17], resulting in the loss of emission area and thus reducing the output efficiency. In addition to the normal Ag-base FC LED device structures, there are also DBR-base FC LED device structure that use ITO and interdigitated metal contact for current spreading layers, but these structures bring out degradation of the optical performance, reliability, and lifetime of the optoelectronic device with the high-power input due to the poor current spreading ability of the interdigitated metal contact [18,19,20].

In this paper, we combine the advantages of silver mirror and DBR while tackling the disadvantages of both, and report the demonstration of an FCLED with a novel composite reflection structure (CRS-FCLED) that simultaneously improves the light output power (LOP) and vertical light extraction efficiency. Compared with the commonly used FCLED with a single Ag-mirror layer as the reflective layer, the CRS-FCLED with an Ag/SiO_2_/distributed Bragg reflector (DBR)/SiO_2_ reflective structure exhibited improved reflectance. Further investigation proved that the SiO_2_/DBR/SiO_2_ composite layer covers the sidewall and part of the area of the n-GaN contact orifices that are evenly distributed across the whole area of the LED chips, which improves the light extraction efficiency. When operated under currents of 700, 1000, and 1500 mA, the light outputs of the CRS-FCLEDs are improved by 3.8, 5.1, and 6.3%, respectively, and the corresponding external quantum efficiencies (EQEs) improved by 3.4, 4.7, and 6.0%, respectively. It exhibited markedly smaller optical degradation and thus higher device reliability as compared to t normal Ag-based FCLED. Moreover, the light emission intensity distributions and far-field angular light emission measurements proved that the intensity of the emission light from the CRS-FCLEDs is significantly increased, especially in the vertical direction, which is advantageous for high-power field applications, such as headlamps for automobiles.

## 2. Materials and Methods

The LED samples were grown on the c-plane of the patterned sapphire substrate using the metal–organic chemical vapor deposition (MOCVD) method. The epitaxial structures of the LED from the bottom to the top were as follows: a 20 nm AlN buffer was deposited by reactive sputtering at 300 ℃, which can be as a buffer layer and interlayer in order to favor an oriented growth and improve the GaN crystal quality [21,22], a 3-μm undoped GaN layer, a heavily Si high doping n-GaN layer with a thickness of 2.5 μm, an InGaN/GaN superlattice structure with a thickness of 120 nm that can act as a strain release layer, five pairs of InGaN/GaN multiple quantum wells (MQWs) with a thickness of 30 nm, a 40-nm low temperature p-GaN layer, a 48-nm p-AlGaN/GaN electron blocking layer, and a 110-nm Mg- doped p-GaN layer. The dimensions of the CRS-FCLED are 1400 × 1400 μm^2^ for high-power applications.

**Figure 1:**
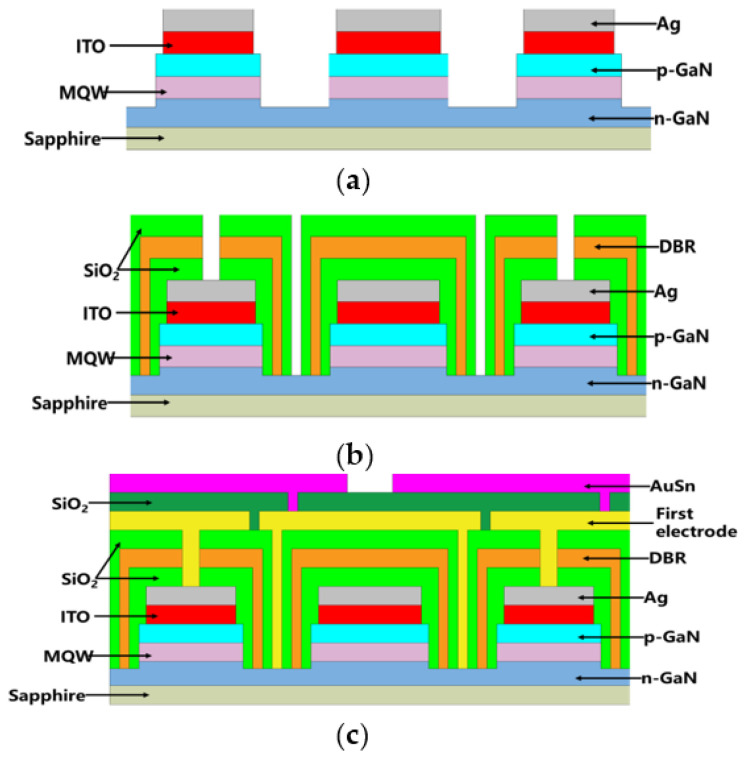
Main fabrication process of the CRS-FCLED. (**a**) a layer of Ag (120 nm) is sputtered on top of the ITO that acts as the reflecting lay-er; (**b**) the p-GaN contact orifices and n-GaN contact orifices were fabricated by ICP etching with a CF4/O2 gas mixture as the etching gas source; (**c**) an AuSn alloy solder layer was deposited by thermal evaporation.3. Results and Discussion.

The main fabrication processes of the CRS-FCLED were shown in Figure 1. The detailed processing steps are as follows: the n-GaN contact orifices are fabricated by an inductively coupled plasma (ICP) etching method with a BCl_3_/Cl_2_ mixture gas. A 10-nm ITO contact layer was then deposited on the p-GaN by the magnetron sputtering method, followed by annealing in N_2_ ambient at 550 °C to improve the contact properties. Subsequently, a layer of Ag (120 nm) is sputtered on top of the ITO that acts as the reflecting layer [Figure 1a]. For the fabrication of the CRS structure, a layer of SiO_2_ (300 nm) was first deposited on top of the Ag-mirror layer, after which an insulating DBR structure with 20 pairs of periodically arranged SiO_2_ and TiO_2_ layers (82.3 nm/40.5 nm) is deposited on the top of the SiO_2_ layer and partially filled the via orifices. Then, a 300-nm SiO_2_ layer is deposited on top of the DBR structure for better passivation protection. Finally, the p-GaN contact orifices and n-GaN contact orifices were fabricated by ICP etching with a CF_4_/O_2_ gas mixture as the etching gas source [Figure 1b]. After the fabrication of the CRS structure, the first electrode layers of Cr (0.5 nm)/Al (1 µm)/Cr (40 nm)/Pt (0.2 µm) were deposited on the DBR and filled the p-contact orifices and n-contact orifices. A SiO_2_ insulating layer with a thickness of 1.2 µm is deposited on the top of the first electrode layer grown by plasma-enhanced chemical vapor deposition, with the interconnected orifices formed by the buffer oxide etchant (BOE) wet etching method. Finally, an AuSn alloy solder layer was deposited by thermal evaporation to meet the welding reliability requirements for high power applications [Figure 1c].

The electrical and optical properties of the FCLEDs were characterized by an auto-matic wafer measurement system and integrating sphere (Everfine, CAS-140D, Hang-zhou, China). The cross-sectional images were analyzed by the scanning electron micro-scope (ZEISS, ∑IGMA 300, Tokyo, Germany). The reflectance of mirrors were measured by ultraviolet spectrophotometer (Hitachi, UH4150, Tokyo, Japan). A 50-W tungsten halogen lamp and a monochromator were used for measuring the reflectance of the mirror. The in-cident angle for the ultraviolet spectrophotometer was 90°. The light emission patterns were analyzed using a light intensity distribution tester (Everfine, LED626, Hangzhou, China). The reliability was tested using reliability test system (Everfine, NK -1020, Hangzhou, China).

## 3. Results and Discussion

### 3.1. High Reflectivity Layers Selection

**Figure 2:**
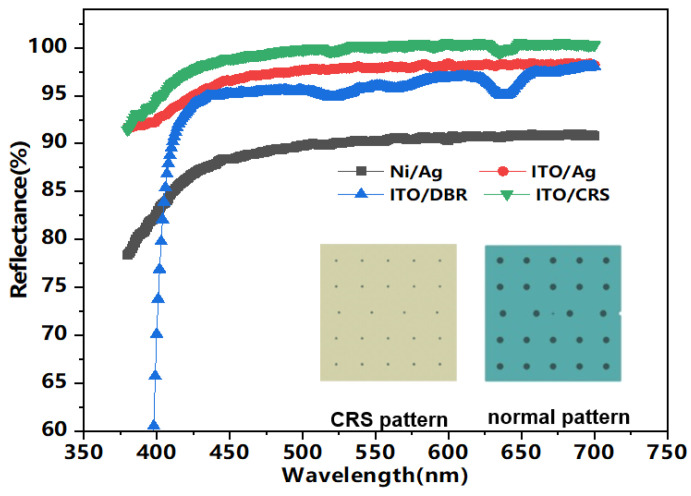
Reflectance of Ni/Ag, ITO/Ag, ITO/DBR, and ITO/CRS films (inset: normal pattern and CRS pattern).

The p-type ohmic contact electrodes of FCLEDs should have high reflectivity and low contact resistance. To realize this, metallic and DBR mirrors can be used as highly reflective layers in flip chips owing to their high reflectance in the visible wavelength range [23]. Further, ITO was sandwiched between the reflection layer and the p-GaN to decrease the p-type contact resistance. On the other hand, DBR, instead of metallic mirrors, can be used as reflection layer [24]. For comparing the reflectance, Ni (1 nm)/Ag (150 nm), ITO (10 nm)/Ag (150 nm), ITO (10 nm)/ DBR (2.1 μm), and ITO (10 nm)/ Ag (150 nm)/ SiO_2_ (300 nm)/ DBR (2.1 μm)/ SiO_2_ (300 nm) films were deposited on the quartz glass. To simulate the actual reflection of the chip, all the layers were patterned by a photoresist mask and etched by ICP etching with a CF_4_/O_2_ gas mixture. The reflectance as a function of wavelength is shown in Figure 2.

At the wavelength of 450 nm, the measured reflectance of Ni/Ag, ITO/Ag, ITO/DBR, and ITO/CRS were 88.38, 96.5, 95.13, and 98.8%, respectively. Ni/Ag exhibits the lowest reflectance due to strong absorption of light by the underlying Ni layer. Compared with Ni/Ag, ITO/DBR showed a higher reflectivity, but the DBR had a very low reflectivity in partial wavelength, and the reflective spectrum was blue shifted toward the short wavelength when the incident angle of light was increased [25]. Further, the higher reflectance of ITO/CRS films in visible light than that of ITO/Ag films and ITO/DBR indicates that ITO/CRS films can efficiently substitute the normal ITO-Ag reflection systems.

### 3.2. Cross-Sectional SEM Image

**Figure 3:**
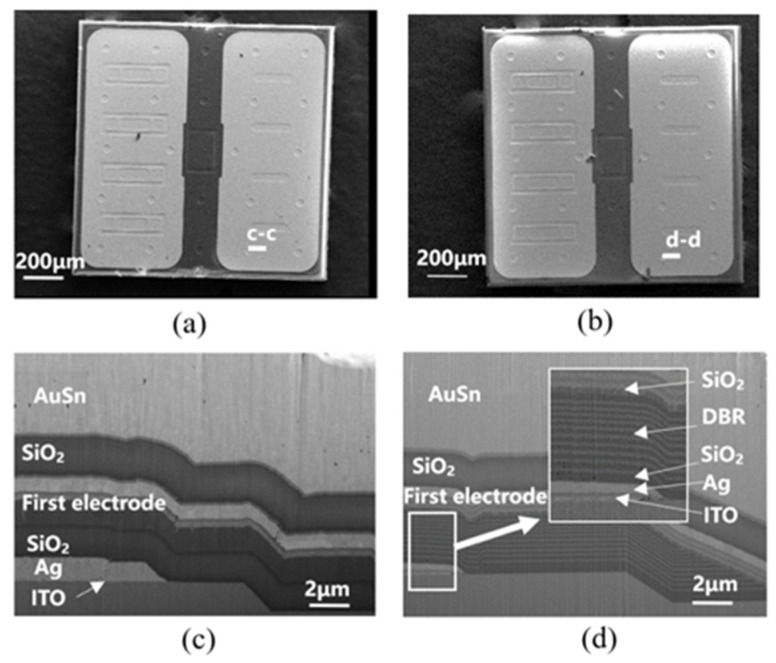

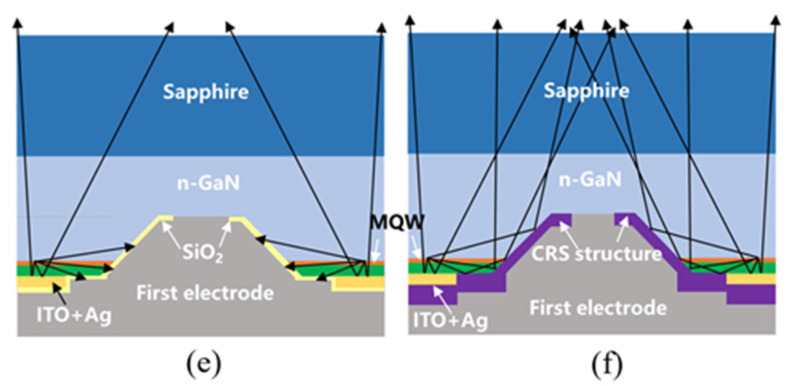
(**a**) Top-view SEM image of normal Ag-based FCLED. (**b**) Top-view SEM image of CRS-FCLED. (**c**) Cross-sectional SEM image of normal Ag-based FCLED milled by FIB along c-c direction. (**d**) Cross-sectional SEM image of CRS-FCLED milled by FIB along the d-d direction. (**e**) Schematic illustration of the light extraction of normal Ag-based FCLEDs. (**f**) Schematic illustration of the light extraction of CRS-FCLEDs.

Figure 3a,b shows a top-view scanning electron microscope (SEM) image of the normal Ag-based FCLED and CRS-FCLED, respectively. Their layouts are nearly identical except for the reflective layer structure. The n-contact orifices are evenly distributed over the entire area of the LED chip to enhance current spreading. The cross-sectional SEM images of both LEDs are shown in Figure 3c,d, and the corresponding schematic illustrations are shown in Figure 3e,f. As shown in Figure 3c,e, the diameter of the n-contact orifices in the Ag layer is larger than that of the n-GaN contact orifices in the normal Ag-based FCLEDs, to avoid Ag migration on forward current aging or bulk leakage by electrode destruction near the V-pit defect region [26]. However, this diameter mismatch can form an emission loss area. The light emitted from the sidewall of each n-GaN contact orifice can be absorbed by n-pad metals with low reflectance, such as Cr and Au, which can decrease the light extraction efficiency. This emission loss phenomenon can be effectively reduced in CRS-FCLEDs. As shown in Figure 3d,f, at the edge of the Ag mirror, the DBR mirror continues to extend the reflection area, covering the upper and lower mesa gentle slope and the n-via area to maximize the reflection area. As a result, CRS-FCLEDs can achieve improved reflection performance compared with normal Ag-based FCLEDs.

### 3.3. Light Emission Distributions

**Figure 4:**
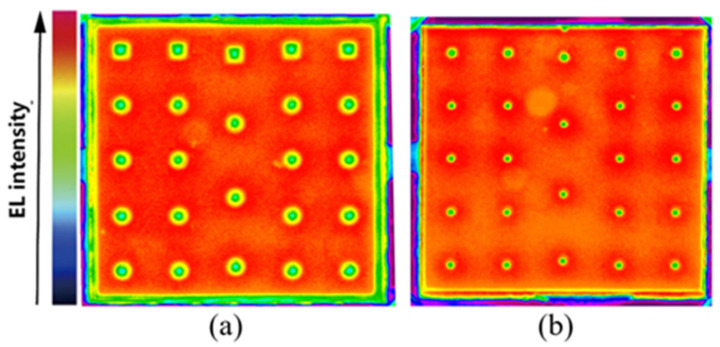
(**a**) Light emission distributions in the normal Ag-based FCLED. (**b**) Light emission intensity distributions in the CRS-FCLED.

The light emission intensity distributions of the normal Ag-based FCLEDs and CRS-FCLEDs, when driven by an injection current of 1500 mA [Figure 4], shows that the total emission region of the CRS-FCLEDs is larger, owing to the increased reflective area at the sidewall and partial area of the n-GaN contact orifices. Moreover, the emission intensity in the emission region around the n-GaN contact orifices is explicitly higher than that in other emission regions. It has been reported that the number of photons generated in the active region around the electrode is significantly higher than that in other regions of the LED because of the current crowding effect around the electrode [27]. Therefore, even though the increased total reflective area of the CRS-FCLEDs is negligible, the light extraction efficiency can be significantly improved because the number of photons generated around the electrodes is considerably higher than that in other emission regions.

Another issue that the uniformity of current spreading on the thermal management is extremely important for the high-power applications. The reason why CRS-FCLED has A better light emission intensity distributions can be explained by the better current distribution. Further indicating that the CRS-FCLED has a more uniform temperature distribution due to the uniform current spreading [28].

### 3.4. Characteristic Curve

**Figure 5:**
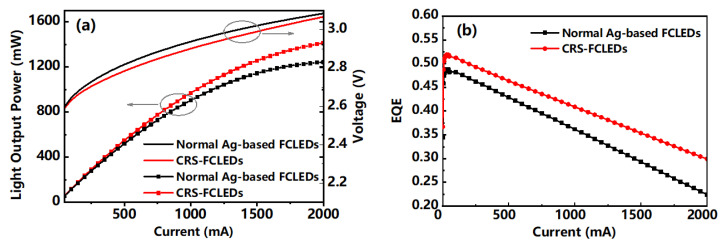
(**a**) Forward voltage and LOP versus injection current for normal Ag-based FCLED and CRS-FCLED. (**b**) EQE versus injection current for normal Ag-based FCLED and CRS-FCLED.

The dependence of the forward voltage and LOP versus injection current for the normal Ag-based FCLED and CRS-FCLED are shown in Figure 5a. The current-voltage curves of the two LEDs are nearly identical, indicating that both have similar p-type and n-type contact spreading resistance [29]. As the injection current increased, the output power of the CRS-FCLEDs showed better performance. At 350 mA, the LOP of the CRS-FCLED is 3.8% higher, which is further increased to 5.1% at 1000 mA. Finally, at 1500 mA, the LOP of the CRS-FCLED is 6.3% higher than that of the normal Ag-based FCLED. Meanwhile, the EQE of the CRS-FCLEDs improved by 3.4, 4.7, and 6.0% at 350, 1000, and 1500 mA, respectively [Figure 5b]. The improvements can be attributed to the increased high reflection area, which helps to increase the light extraction efficiency. In addition, the degree of enhancement is larger at high injection currents, which can be explained by the better current distribution of the CRS structure.

### 3.5. Optical Degradation Reliability

**Figure 6:**
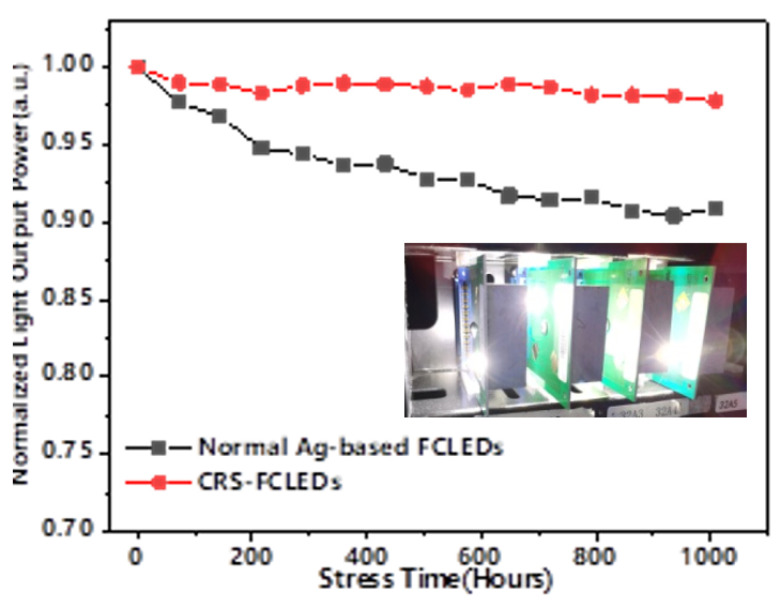
Optical degradation of CRS-FCLED and normal Ag-based FCLED during high-temperature operation life test. (inset: Reliability testing system).

Moreover, as shown in Figure 6, the optical degradations of the CRS-FCLED and normal Ag-based FCLED at 85 °C were also investigated using an injection current of 1500 mA. After high temperature operation life test (1000 h), the light output power of CRS-FCLED decreased by 3.07%, whereas that of the normal Ag-based FCLED decreased by 9.92%. Clearly, the CRS-FCLED exhibits significantly smaller optical degradation and thus, offers a higher device reliability as compared to the normal Ag-based FCLED.

**Figure 7:**
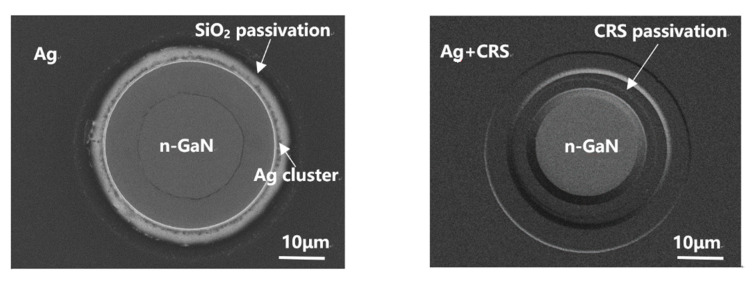
SEM top view of FCLED after forward current aging with (**a**) SiO_2_ passivation layer; (**b**) CRS passivation layer.

To investigate the optical degradations origin, SEM observations were performed for the FCLEDs after forward current aging, as shown in Figure 7a,b. Notably, after high temperature operation life test (1000 h), the Ag-based FCLED sample with SiO_2_ passivation layer showed many metallic clusters on adjacent mesa surface, indicating a migration of Ag to n-contact side. Interestingly, no Ag cluster was observed in the sample with CRS passivation layer. Therefore, current crowding across the thick p-reflective electrode adjacent to the n-electrode would result in surface leakage and output drop because of Ag migration on forward current aging, the forward current aging resulted in a noticeable output degradation in Ag-based FCLEDs due to the surface migration of Ag [30,31]. The CRS structure covered by the SiO_2_/DBR/SiO_2_ passivation layers consisting of three dielectric stack layers is found to be effective in suppressing the Ag migration. In addition, the mentioned structure is considerably effective in passivating the exposed surfaces of ITO and n-GaN layers after ICP etching, resulting in decreasing the trap density near the surface, minimizing the leakage current through the surface of the LED. Ag migration protection will determine the reliability of the flip-chip in high current injection conditions. In contrast with the normal Ag-based FCLED with a SiO_2_ passivation layer that is less than 1 µm thick, the CRS structure covers the p-reflective electrode and n-electrode with over 3-µm SiO_2_/DBR/SiO_2_ sandwich passivation layer, which further effectively blocks the path of Ag migration to the n-electrode.

### 3.6. Far-Field Radiation Pattern

**Figure 8:**
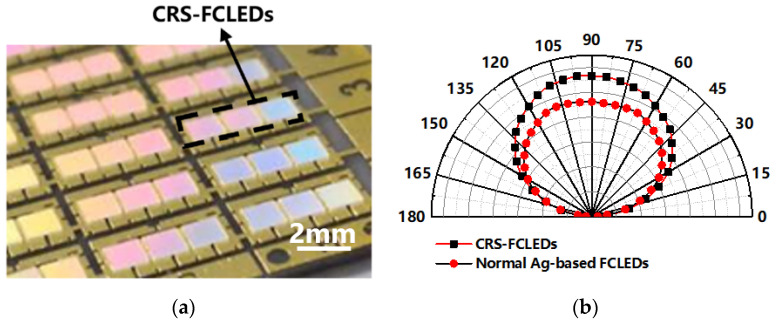
(**a**) LED high power module with dense matrix package. (**b**) Far-field radiation pattern of CRS-FCLED and normal Ag-based FCLED at 1500 mA.

Figure 8a shows the LED module fabricated with our CRS-FCLEDs for use in a high-power application. All the LED chips were packaged in an AlN ceramic matrix with high thermal conductivity. For high-power applications, the LED module must have a high packaging density to obtain the maximized optical density per unit area, with the distance between LED modules designed to be minimized within a short range of only 100–200 μm. However, high losses occur at large emission angles due to the narrow distance between chips, and this is not beneficial for the light output of the LED module. Figure 8b shows the far-field angular light emission patterns of the normal Ag-based FCLEDs and CRS-FCLEDs. The operational current is 1500 mA for both LEDs, which is in accordance with the normal working conditions of the headlight module. Compared with the normal Ag-based FCLEDs, the intensity of the emission light from the CRS-FCLEDs is significantly increased, especially in the vertical direction, and the intensity of the emission light in the large angle direction is rarely increased. This result can be well explained by the additional reflection area of the CRS-FCLEDs. As the location of the additional reflective area is on the sidewall and covers part of the n-GaN contact orifices, the photons emitted from the side wall can be extracted with the emission angle changing in the vertical direction. As a result, the CRS-FCLED achieved the unique characteristic of strengthened light output in the vertical direction.

## 4. Conclusions

In summary, FCLEDs with a novel composite reflection structure of Ag/SiO_2_/DBR/SiO_2_ were fabricated, which simultaneously improved the light extraction efficiency and light output in the vertical direction. Compared with the conventional FCLEDs with a single Ag mirror as the reflective layer, the reflective area of the CRS-FCLEDs is increased because the sidewall and part of the n-GaN contact orifices had been covered by the highly reflective SiO_2_/DBR/SiO_2_ sandwich structure. As a result, the LOP of the CRS-FCLEDs increased by 6.3% at an operational current of 1500 mA, while the corresponding EQE was improved by 6.0%, and it exhibited markedly smaller optical degradation and thus higher device reliability as compared to t normal Ag-based FCLED. Moreover, the light emission intensity distributions and the far-field angular light emission pattern proved that it exhibited a higher light output in the vertical direction, suggesting that the CRS-FCLEDs have potential in high power applications.

## References

[1] Bhardwaj J., Cesaratto J.M., Wildeson I.H., Choy H., Tandon A., Soer W.A., Schmidt P.J., Spinger B., Deb P., Shchekin O.B. (2017). Progress in high-luminance LED technology for solid-state lighting. Phys. Status Solidi A.

[2] Ying S.P., Shen W.B. (2015). Thermal analysis of high-power multichip COB light-emitting diodes with different chip sizes. IEEE Trans. Electron Devices.

[3] Tao X.H. (2015). Performance characterization and theoretical modeling of emitted optical power for high-power white-LED devices. IEEE Trans. Electron Devices.

[4] Li J.-S., Tang Y., Li Z.-T., Li J.-X., Ding X.-R., Yu B.-H., Yu S.-D., Ou J.-Z., Kuo H.-C. (2021). Toward 200 lumens per watt of quantum-dot white-light-emitting diodes by reducing reabsorption loss. ACS Nano.

[5] Lee G.J., Hong I.Y., Kim T.K., Park H.J., Oh S.K., Cha Y.-J., Park M.J., Choi K.J., Kwak J.S. (2019). Design of ITO/SiO_2_/TiO_2_ distributed Bragg reflectors as a p-type electrode in GaN-based flip-chip light emitting diodes. Appl. Surf. Sci..

[6] Chong W.C., Lau K.M. (2014). Performance enhancements of flip-chip light-emitting diodes with high-density n-type point-contacts. IEEE Electron Device Lett..

[7] Zhou S., Zheng C., Lv J., Gao Y., Wang R., Liu S. (2017). GaN-based flip-chip LEDs with highly reflective ITO/DBR p-type and via hole-based n-type contacts for enhanced current spreading and light extraction. Opt. Laser Technol..

[8] Li Z.T., Tang Y., Li J., Ding X.R., Yan C.M., Yu B.H. (2018). Effect of flip-chip height on the optical performance of conformal white-light-emitting diodes. Opt. Lett..

[9] Liu N., Yi X., Wang L., Sun X., Liu L., Liu Z., Wang J., Li J. (2015). Light extraction improvement of blue light-emitting diodes with a metal-distributed Bragg reflector current blocking layer. Appl. Phys. A.

[10] Ding X.R., Tang Y., Li Z.T., Li J.S., Xie Y.X., Lin L.W. (2017). Multichip LED modules with V-groove surfaces for light extraction efficiency enhancements considering roughness scattering. IEEE Trans. Electron Devices.

[11] Li X.H., Zhu P., Liu G., Zhang J., Song R., Ee Y.K., Kumnorkaew P., Gilchrist J.F., Tansu N. (2013). Light extraction efficiency enhancement of III-nitride light-emitting diodes by using 2-D close-packed TiO2 microsphere arrays. J. Disp. Technol..

[12] Ge D., Huang X., Wei J., Qian P., Zhang L., Ding J., Zhu S. (2019). Improvement of light extraction efficiency in GaN-based light-emitting diodes by addition of complex photonic crystal structure. Mater. Res. Express.

[13] Matioli E., Weisbuch C. (2011). Direct measurement of internal quantum efficiency in light emitting diodes under electrical injection. J. Appl. Phys..

[14] Park J.-S., Han J., Han J.-W., Seo H., Oh J.-T., Seong T.-Y. (2013). Improving the output power of near-ultraviolet InGaN/GaN-based light emitting diodes by enhancing the thermal and electrical properties of Ag-based reflector. Superlattices Microstruct..

[15] Lee J.-H., Hwang S.-M., Kim N.-S., Lee J.-H. (2010). InGaN-based high-power flip-chip LEDs with deep-hole-patterned sapphire substrate by laser direct beam drilling. IEEE Electron Device Lett..

[16] Song J.O., Kwak J.S., Park Y., Seong T.-Y. (2005). Ohmic and degradation mechanisms of Ag contacts on p-type GaN. Appl. Phys. Lett..

[17] Son J.H., Song Y.H., Yu H.K., Lee J.-L. (2009). Effects of Ni cladding layers on suppression of Ag agglomeration in Ag-based Ohmic contacts on p-GaN. Appl. Phys. Lett..

[18] Zhou S., Liu X., Yan H., Chen Z., Liu S. (2019). Highly efficient GaN-based high-power flip-chip light-emitting diodes. Opt. Express.

[19] Zhou S.J., Liu X.T., Gao Y., Liu Y., Liu M.L., Liu Z.Y., Gui C.Q., Liu S. (2017). Numerical and experimental investigation of GaN-based flip-chip light-emitting diodes with highly reflective Ag/TiW and ITO/DBR Ohmic contacts. Opt. Express.

[20] Shiu G.Y., Chen K.T., Fan F.H., Huang K.P., Hsu W.J., Dai J.J., Lai C.F., Lin C.F. (2016). InGaN light-emitting diodes with an embedded nanoporous GaN distributed bragg reflectors. Sci. Rep..

[21] Polyakov A.Y., Smirnov N.B., Govorkov A.V., Belogorokhov I.A., Scherbatchev K.D., Bublik V.T., Avdeev O.A., Chemekova T.Y., Mokhov E.N., Nagalyuk S.S. (2011). Structural and electric properties of AlN substrates used for LED heterostructures’ growth. Russ. Microelectron..

[22] Mariello M., Fachechi L., Guido F., De Vittorio M. (2021). Conformal, ultra-thin skin-contact-actuated hybrid piezo/triboelectric wearable sensor based on AlN and parylene encapsulated elastomeric blend. Adv. Funct. Mater..

[23] Lin T.H., Wang S.J., Tu Y.C., Hung C.H., Yu T.H. (2016). Improving the performance of power GaN-based thin-film flip-chip LEDs through a two fold roughened surface. Mater. Sci. Semicond. Process..

[24] Genç M., Sheremet V., Elçi M., Kasapoğlu A.E., Altuntaș İ., Demir İ., Eğin G., İslamoğlu S., Gür E., Muzafferoğlu N. (2019). Distributed contact flip chip InGaN/GaN blue LED; comparison with conventional LEDs. Superlattices Microstruct..

[25] Zhou S.J., Xu H.H., Liu M.L., Liu X.T., Zhao J., Li N., Liu S. (2018). Reflector on electrical and optical properties of GaN-based flip-chip light-emitting diodes. Micromachines.

[26] Jeong S., Kim M.S., Lee S.-N., Kim H. (2019). Forward and reverse current aging of GaN-based light-emitting diodes fabricated with Ag-based reflective electrodes. Mat. Sci. Semicon. Proc..

[27] Kim H., Cho J., Park Y., Seong T.Y. (2008). Leakage current origins and passivation effect of GaN-based light emitting diodes fabricated with Ag p-contacts. Appl. Phys. Lett..

[28] Horng R.H., Chuang S.H., Tien C.H., Lin S.C., Wuu D.S. (2014). High performance GaN-based flip-chip LEDs with different electrode patterns. Opt. Express.

[29] Sheu J.K., Hung I.-H., Lai W.C., Shei S.C., Lee M.L. (2008). Enhancement in output power of blue gallium nitride-based light-emitting diodes with omnidirectional metal reflector under electrode pads. Appl. Phys. Lett..

[30] Chen H.T., Cheung Y.F., Choi H.W., Tan S.C., Hui S.Y. (2015). Reduction of thermal resistance and optical power loss using thin-film light-emitting diode (LED) structure. IEEE Trans. Ind. Electron..

[31] Liu X., Li N., Hu J.F., Gao Y.L., Wang R.Q., Zhou S.G. (2018). Comparative study of highly reflective ITO/DBR and Ni/Ag ohmic contacts for GaN-based flip-chip light-emitting diodes. Solid State Sci. Technol..

